# Investigation of the Azorean *Camellia sinensis* Processing Conditions to Maximize the Theaflavin 3,3′-di-*O*-Gallate Content as a Potential Antiviral Compound

**DOI:** 10.3390/antiox11061066

**Published:** 2022-05-27

**Authors:** Lisete Paiva, Elisabete Lima, Madalena Motta, Massimo Marcone, José Baptista

**Affiliations:** 1 Gorreana Tea Plantation, Gorreana, 9625-304 Maia, Portugal; lisete.s.paiva@uac.pt (L.P.); gorreanazores@gmail.com (M.M.); 2 Department of Physics, Chemistry and Engineering (DCFQE), Faculty of Science and Technology, University of Azores, 9500-321 Ponta Delgada, São Miguel, Azores, Portugal; jose.ab.baptista@uac.pt; 3 Institute of Agricultural and Environmental Research and Technology (IITAA), University of Azores, 9700-042 Angra do Heroísmo, Terceira, Azores, Portugal; 4 Department of Food Science, University of Guelph, Guelph, ON N1G 2W1, Canada; mmarcone@uoguelph.ca

**Keywords:** coronavirus, COVID-19, tea polyphenolic components, theaflavin-3,3′-di-*O*-gallate, Azorean *Camellia sinensis*, tea plantation zones, novel antiviral black tea, RP-HPLC/PDAD analysis

## Abstract

The molecular constituents of *Camellia sinensis*, in particular epigallocatechin-3-*O*-gallate (EGCG) and, more remarkably, the galloylated theaflavins, mainly theaflavin-3,3′-di-*O*-gallate (TF-3,3′-DG), have been reported to inhibit SARS-CoV-2 3-chymotrypsin-like protease (3CL^pro^), an enzyme required for the cleavage of its polyproteins, to produce vital individual functional proteins for viral cell replication. Our results for total catechin content revealed the values of 174.72, 200.90, and 211.75 mg/g dry weight (DW) in spring, and the values of 183.59, 191.36, and 215.09 mg/g DW in summer, for tea plantation zones 1, 2, and 3, respectively. For the TF-3,3′-DG content, the values of 2.68, 1.13, and 3.72 mg/g DW were observed in spring, and the values of 3.78, 2.06, and 8.91 mg/g DW in summer for zones 1, 2, and 3, respectively. In the same zone, different contents of TF-3,3′-DG were observed across plucking months of April, June, and August, with values of 1.13, 2.77, and 4.18 mg/g DW, respectively, showing higher values in summer. Different values of TF-3,3′-DG contents were also observed in the same tea plantation zone but from different plant parts, revealing higher values in the bud and the first and second leaves (3.62 mg/g DW) and lower values in the third and fourth leaves (1.14 mg/g DW). The TF-3,3′-DG content increased from 3.31 to 4.98 mg/g DW with increased fermentation time from 1 to 3 h, respectively, and increased for lower temperature and longer fermentation time. The aim of this study was to investigate the processing conditions that lead to maximum TF-3,3′-DG content and, given its potential impact as an inhibitor of the 3CL^pro^ enzyme, to create a novel antiviral Azorean black tea.

## 1. Introduction

Following water, tea, a drink made from *Camellia sinensis* (L.) tea leaves, is one of the oldest and most widely consumed non-alcoholic beverages worldwide due to its stimulant properties, low retail price, pleasant sensory characteristics, and scientifically proven beneficial effects on human health [1,2]. The tea plant, native to Southeast China, gradually expanded into many tropical and subtropical countries, and since the last decade of the 19th century, it is also commercially produced in one unique place in Europe, the volcanic São Miguel Island of the Azores Archipelago (Portugal) [3].

During *C. sinensis* tea production, tea is effectively separated into non-fermented, semi-fermented, and fermented tea types. Enzymes such as polyphenol oxidase (PPO) and peroxidase (POD), which are present in plant leaves, are inactivated by being boiled, steamed by vapor, or subjected to electrical heat or microwaves for non-fermented green tea production. However, one of the most consumed teas in the world (75%) is black tea, in which the catechins are oxidized during the fermentation process. Both green tea, which contains approximately 70 different tea polyphenols (TPs), particularly catechin (C), epicatechin (EC), epigallocatechin (EGC), epicatechin-3-*O*-gallate (ECG), and epigallocatechin-3-*O*-gallate (EGCG), and black tea, which contains hydrolysable tannins and secondary polyphenols including theaflavins, theaflagallins, and theasinensins, are reported to offer several benefits for human health [4]. The TPs, particularly the group of catechins (flavan-3-ols), are considered key contributors to the protective effects that green tea offers against several diseases (e.g., cancer, cardiovascular disease, type 2 diabetes, and neurodegenerative diseases), as well as the aging process, and recently have been used against several viruses and found to be a potential treatment option, avoiding the side effects of synthetic drugs [5,6]. Tea plants also synthesize volatile terpenoids and phenolics in the form of glycosides. These hydrolytic products, released during tea processing, provide different types of teas with a variety of characteristic flavors and pleasant sensory aromas. However, it is well established that the composition of commercial teas can be significantly affected by many factors, such as the plant variety, origin of tea production, climate, genetic strain, leaf age, and plucking season, as well as the agronomic management, tea processing, and storage conditions [7,8].

The causative agent of the ongoing coronavirus disease 2019 (COVID-19) pandemic, referred to as severe acute respiratory syndrome coronavirus 2 (SARS-CoV-2), is a positive-sense RNA single-stranded virus, and is spreading at a frightening rate, leading to an enormous program of vaccines, but additional years will be required to achieve complete immunity [5]. On the other hand, it is expected that other variants or coronavirus subfamilies will emerge in the near future. Therefore, antiviral drugs should be urgently developed to treat or mitigate the negative effects of COVID-19. Several antiviral drugs are under clinical trials, and amongst the drugs being tested, chloroquine, hydroxychloroquine, remdesivir, and new chemical molecules have received a lot of attention within the scientific community [9]. Unfortunately, due to possible side effects, higher doses of these drugs cannot be administered.

As we currently know, EGCG and theaflavins are polyphenolic components found abundantly in green and black teas, respectively, with an enormous array of health benefits. Their antiviral activities have been reported against various viral infections, acting at different stages of the viral cycle [10]. On the other hand, 3-chymotrypsin-like protease (3CL^pro^), also referred to as M^pro^, is a vital proteolytic enzyme in SARS-CoV-2 due to its important function in viral cell replication [5,6]. Recently, some studies have demonstrated that EGCG and all the theaflavins are found to inhibit M^pro^ and exhibit a good binding efficacy with the major receptor binding domain (RBD) of the surface (S) glycoprotein in SARS-CoV-2 by forming hydrophobic interactions as well as hydrogen bonds with some sites of this glycoprotein [10]. Ohgitani [5] reported that viruses treated with galloylated theaflavins, particularly theaflavin-3,3′-di-*O*-gallate (TF-3,3′-DG), at 100 µM showed less than 1/10.000 infectivity compared with untreated viruses.

Recently, studies have demonstrated that the SARS-CoV-2 S glycoprotein, containing 1273 amino acids, has 22 N-linked and 2 O-linked glycan sites, which may have a significant effect in protein folding, receptor binding, and immune escape [9]. Additionally, theaflavins, particularly TF-3,3′-DG, are known to provide RNA-dependent RNA polymerase (RdRp) inhibition and angiotensin-converting enzyme 2 (ACE2) binding activity. Several studies have corroborated that, in SARS-CoV-2 infection, the viral S protein receptor-binding domain binds to ACE2 to enter the host cells [10].

**Figure 1:**
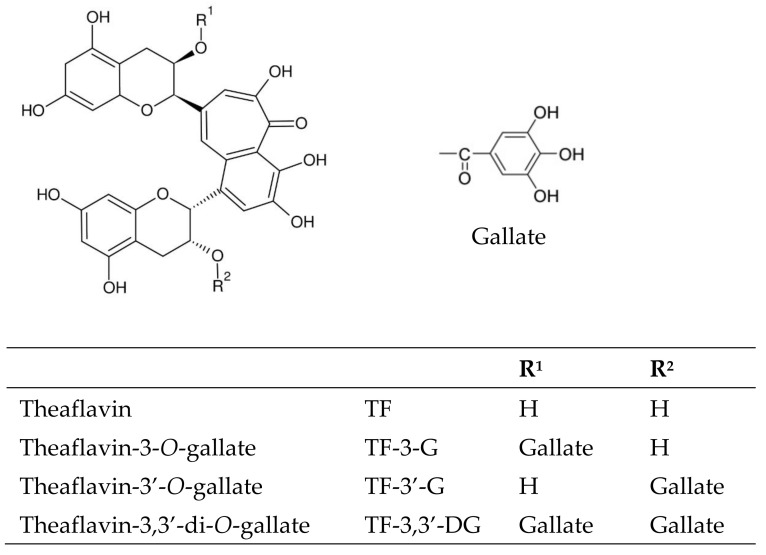
Chemical structures of theaflavins (TF, TF-3-G, TF-3′-G, and TF-3,3′-DG) found in black tea.

In recent years, the theaflavins, namely theaflavin (TF), theaflavin-3-*O*-gallate (TF-3-G), theaflavin-3′-*O*-gallate (TF-3′-G), and TF-3,3′-DG (Figure 1), have attracted considerable attention within the scientific community as they have been shown to have numerous physiological actions, including antioxidant [11,12], anti-atherosclerotic [13,14], anti-inflammatory [15], anticancer [16,17,18], and antiviral [19,20] effects. Since EGCG and theaflavins are from natural sources and consumed regularly by the world’s population, particularly in Asian countries, investigating the antiviral properties of these compounds against COVID-19 could represent a significant step forward in the search for new treatments effective against this latest pandemic disease [5,6].

Taking all of the in vitro reported studies and the in vivo results comparing COVID-19 mortality (per million population) in countries with low tea consumption and countries with high tea consumption, along with our experience in studying the chemical composition of samples from different zones of tea plantation, the objective of our study was to investigate the effect of Azorean black tea processing conditions: this included the effect of the fermentation time and the temperature of fermentation, the effect of PPO and POD on the tea fermentation process, the variability in theaflavin content in different zones of tea plantation, and the seasonality effect, in order to increase the content of Azorean theaflavins, particularly TF-3,3′-DG, and, consequently, to create an antiviral novel Azorean black tea, that according to several studies shows an impact on the reduction/inhibition of the SARS-CoV-2 infectivity.

## 2. Material and Methods

### 2.1. Chemicals and Reagents

Catechins, namely, (+)-catechin (C, 98%—C1251), (−)-epicatechin (EC, 98%—E4018), (−)-epigallocatechin (EGC, 98%—E3768), (−)-epigallocatechin-3-*O*-gallate (EGCG, 95%—E4143), (−)-epicatechin-3-*O*-gallate (ECG, 98%—E3893), (+)-gallocatechin (GC, 98%—G6657) and (−)-gallocatechin-3-*O*-gallate (GCG, 98%—G6782), caffeine (CAF, 99%—C0750), gallic acid (98%—G7384), theaflavin (TF), theaflavin-3-*O*-gallate (TF-3-G), theaflavin-3′-*O*-gallate (TF-3′-G), and theaflavin-3,3′-di-*O*-gallate (TF-3,3′-DG), were obtained from Sigma–Aldrich (St. Louis, MO, USA). Orthophosphoric acid was obtained from E. Merck (Darmstadt, Hessen, Germany). Acetonitrile, methanol, chloroform, and ethyl acetate, of HPLC-grade, were obtained from Riedel-de Häen (Aktiengesellschaft, Seelze, Germany). Glass distilled water that was deionized with the Millipore Milli-Q purification system (Millipore, Bedford, MA, USA) was used throughout all the experiments.

### 2.2. Sample Origin, Withering, and Drying Methodologies

The fresh *C. sinensis* leaf is unusually rich in polyphenols (catechins), which remain practically unchanged in commercial green tea when a good manufacturing process is employed, except for a few enzymatically catalyzed reactions that occur rapidly following plucking. In the process of black tea manufacture, catechins are oxidized followed by a series of chemical condensations, forming theaflavins and thearubigins. *C. sinensis* samples from tree different zones (zone 1—Lat: 37.819027, Long: −25.402379 and altitude 205 m; zone 2—Lat: 37.812773, Long: −25.403037 and altitude 235 m; and zone 3—Lat: 37.809894, Long: −25.403627, and altitude 360 m above sea level) of Gorreana Tea Plantation were used. The *C. sinensis* plants grow in volcanic soil, which has an average pH = 5.6 (range of 4.1–6.3) and is rich in basic elements: N = 0.17 g/100 g, P = 58 mg/Kg, K = 0.49 meq/100 g, and Mg = 0.50 meq/100 g, expressed per weight of dried soil (average data obtained from Gorreana Tea Plantation). The fresh tea leaves, under the same processing conditions, were indoor-withered for several hours at 20–25 °C to achieve a relative humidity of 70%. The protocol for the total TPs and CAF extractions was a slightly modified version of the methodology described by Baptista et al. [21]. In black tea production, the tea leaves were oxidized by the PPO, which catalyzes the oxidation of substrates in the presence of oxygen, whereas POD catalyzes the oxidation of substrates in the presence of hydrogen peroxide, providing the red/brown pigments of black tea products, characteristic of the theaflavins and theorubigins produced from their parent catechins. Four types of theaflavins can be obtained by the combination of *C. sinensis* catechins presented in fresh tea leaves as shown:

EC + EGC → TF

EC + EGCG → TF-3-G

ECG + EGC → TF-3′-G

ECG + EGCG → TF-3,3′-DG

### 2.3. Sample Preparation and Extraction Methodology for Crude Catechins and CAF Content

**Figure 2:**
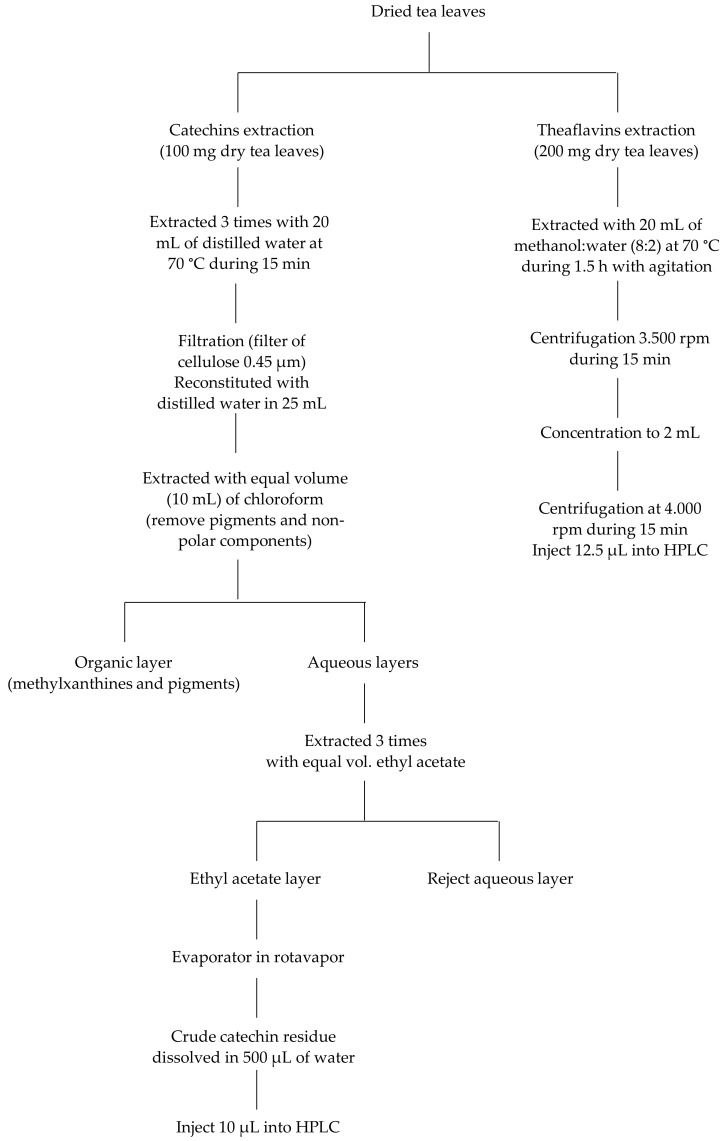
Extraction of catechins and theaflavins from Azorean black tea samples.

By knowing the effect of sample preparation conditions (e.g., extraction time and temperature, solvent type, and solute:solvent ratio) on the tea extraction yield, as well as to simulate the tea infusions used by common people [3,21], our aqueous TP extraction protocol was performed as schematically presented in Figure 2. The dried sample was ground in a mortar to a particle size of 20–30 µm, and the powdered material (1 g) was extracted 3 times with water (20 mL) for 15 min at 70 °C, with stirring at 250 rpm, under an atmosphere of N_2_, to prevent oxidation. The combined extracts solution was filtered under vacuum through a cellulose acetate membrane of 0.45 µm (pore size) to remove particulate matter, then was freeze-dried after vacuum rotary evaporator concentration, and stored for further analysis. The extraction of crude catechins and CAF was performed according to the method published by Baptista et al. [3] with slight modifications. A freeze-dried tea powder (100 mg) was extracted with water using the methodology described above, concentrated in a vacuum rotary evaporator, and reconstituted in 25 mL (volumetric flask) of distilled water. A volume of 10 mL was partitioned with an equal volume of chloroform to remove pigments and other non-polar plant material, and then, the aqueous layer was extracted with equal volume of ethyl acetate to obtain the catechin mixture. The combined extract solution was evaporated in a vacuum rotary evaporator and the crude catechins were dissolved in 500 µL of water, and after being filtered through a 0.45 µm polytetra-fluoroethylene membrane cartridge, 10 µL was injected into high-performance liquid chromatography/photodiode array detection (HPLC/PDAD).

### 2.4. Sample Preparation and Extraction Methodology for Theaflavin Content

Our protocol for the extraction of theaflavins from different tea samples is also shown in Figure 2 and was performed according to the method published by Matsubara et al. [22] with slight modifications. For each tea sample, 200 mg of dry tea leaves was brewed with a 20 mL solution (80% methanol in water) for 1.5 h at room temperature with mild stirring (250 rpm). The tea infusion was filtrated through a 0.45 µm (pore size) cellulose acetate membrane to remove particulate matter. A supernatant volume of 10 mL was concentrated in a rotary evaporator and reconstituted in 2 mL of methanol, and then 12.5 µL was submitted to HPLC analysis.

### 2.5. RP-HPLC Analysis of Catechins, CAF and Theaflavins

The variability of *C. sinensis* samples in terms of the catechin and theaflavin profiles, as well as CAF contents, was determined by RP-HPLC/PDAD, which provides an efficient and high-throughput methodology for separation and quantification of these tea components; this method was already validated in our previous research on tea extracts [3,21]. A Spherisorb ODS2-5 µm (100 × 4.6 mm i.d.) column from LKB (Bromma, Sweden) was used to separate the individual catechins. The mobile phase A was composed of acetonitrile:ethyl acetate:0.1% orthophophoric acid:water (4.25:1:44.75:50, *v*/*v*/*v*/*v*), and mobile phase B was acetonitrile:water (1:1, *v*/*v*). Baseline separation was achieved with a gradient elution as follows: t = 0 min—100% A, t = 10 min—100% A, followed by a linear gradient between phase A and phase B, at an increasing rate of 2% per min of phase B until 20% B was reached, with this composition being maintained until the end of the run at a flow rate of 0.8 mL/min. The column was attached to an Agilent Technologies (Palo Alto, CA, USA) series 1200 Liquid Chromatograph system equipped with a PDAD fixed at 280 nm and was maintained at 30 °C. The total run time was approximately 34 min, and the equilibration time before the following run was 10 min.

For the theaflavin determination, a Hypersil ODS-5 µm (100 × 4.6 mm i.d.) column was used. The mobile phase A was composed of H_2_O: formic acid (99.7:0.3, *v*/*v*) and mobile phase B was methanol:formic acid (99.7:0.3, *v*/*v*). The presence of a low formic acid concentration was necessary to improve the selectivity of the system. The maximal response was achieved with 0.3% formic acid in the two phases of the mobile phase. The separation was achieved by a linear gradient in the following conditions: t = 0 min—30% B, t = 6 min—30% B, t = 40 min—39% B, and t = 50 min—60% B at a flow rate of 1 mL/min. The column was attached to a 1200 Liquid Chromatograph system equipped with a PDAD fixed at 365 nm and was maintained at 40 °C.

The quantitative analyses were performed according to the external standard method using the ChemStation Chromatography Software from Agilent Technologies model 1200 HPLC system (Avandale, PA, USA). The sample concentration was limited to the range of linearity to avoid retention time (RT) shifting, which may occur when the amount of sample approaches the column sample’s load capacity. Peak identification was achieved by RT based on a comparison with the authentic standards. Individual catechins and theaflavins were further confirmed by superimposing the spectrum of each peak with the corresponding authentic standard spectrum. The multilevel working calibration curves at five different concentrations, along with the linear range in µmol/L for the TF-3,3′-DG, were determined, as well as the limits of detection (LOD) and quantification (LOQ), defined as the amount that gives signal-to-noise ratios of 3 and 10, respectively. The results were expressed as mg/g of the sample mass on a dry-weight (DW) basis.

### 2.6. Statistical Analysis

All determinations were performed in triplicate, and the average is expressed as means ± standard deviations (SD). A significance difference was verified by one-way ANOVA on a confidence level of 95% (*p* < 0.05) in SPSS (version 20.0, SPSS Inc., Chicago, IL, USA).

## 3. Results and Discussion

### 3.1. Collection and Processing the Different Azorean C. sinensis Samples and Determination of their Catechin Profiles and CAF Content

Azorean green tea is processed between March and September of each year, and the amount of green TPs is generally 1.4-fold higher in summer than in spring, presumably because of the higher rate of metabolic activities in the young leaves during summer. Approximately 30% of the green tea leaves’ dry weight is TPs, which are the major soluble components in a cup of tea (*C. sinensis*), although variations may be considerable among different batches of tea samples. They exist principally as flavanols (mostly catechins), with the flavonols usually being *O*-glycosides and phenolic acids. Catechins (EGC, C, EC, EGCG, and ECG) have been regarded as the major phenols in almost all kind of teas. The contents of five major catechins in tea infusions were determined by HPLC, and the results are shown in Table 1.

Among all the catechins tested, ECG and EGCG were the two predominant types of catechin in all zones of Azorean tea plantations, accounting for 140.48 and 159.72 for zone 1, 168.34 and 151.87 for zone 2, and 192.75 and 186.25 for zone 3, in mg/g DW for spring and summer samples, respectively, processed under the same conditions. For ECG, an inverse relationship with EGCG was observed, with higher values in summer than in spring for all zones, and the same pattern was also observed for C, with higher values in summer than in spring. On the other hand, Koch et al. [23,24] reported that EGC and EGCG were the two major catechins in several green and black tea samples from different cultivation areas, although they found that the primary catechin in both the green and black teas was not EGCG but EGC. A previous study demonstrated that the concentration of major phenolic compounds including EC, ECG, EGC, and EGCG in black tea infusion is related to the brewing time and that, after 2 min, most phenolics had been extracted. The extract composition did not significantly change at prolonged extraction times (4 min) [17]. Conversely to Zheng et al. [25], who reported higher EGCG content in samples from summer, our results revealed higher EGCG content in the spring samples. The EGCG content was higher in spring zones 2 and 3, with values of 94.71 and 133.64 mg/g DW, respectively, than in summer, which yielded values of 54.23 and 121.30 mg/g DW, respectively. Zone 1 presented lower values in comparison to zones 2 and 3 in the same seasons (67.01 for spring and 54.03 mg/g DW for summer), which can be explained by the lower altitude (205 m) and, consequently, a slightly different climate conditions. In general, the total catechin contents indicated that zone 3 presented higher value, followed by zone 2 and zone 1; however, according to the seasons, spring samples from zone 2 presented slightly higher values than summer samples (200.90 and 191.36 mg/g DW, respectively). Conversely, zones 1 and 3 presented higher values in summer (183.59 and 215.09 mg/g DW, respectively) than in spring (174.72 and 211.75 mg/g DW, respectively), which can be explained by the young tea plants and higher rate of metabolism in summer for zone 1, and the 360 m of altitude for zone 3, which has an effect in soil pH and, consequently, on the enzymatic action of PPO [17].

As stated in Table 1, there was a significant difference among tea samples from different tea plantation zones in terms of the major catechins (a subgroup of flavan-3-ols), epicatechin derivatives (ECDs), and CAF contents. The contents of CAF are at the same level in spring and summer for leaves obtained from zone 1 (16.12 and 16.18 mg/g DW, respectively) and zone 2 (13.67 and 12.27 mg/g DW, respectively), while a significant difference was observed in zone 3 (6.40 and 14.15 mg/g DW) for spring and summer, respectively. Catechin (C) was found to have the lowest content, ranging from 3.94 to 6.69 mg/g DW in zone 2 for spring and summer, respectively, with higher values being observed in summer samples across all zones. The EC ranged from 14.62 to 26.55 mg/g DW in spring for zone 3 and summer for zone 2, respectively. On the other hand, Zhao et al. [12] reported significantly lower values for the catechins with respect to our results, which may be associated with differences in terms of the analytical methodologies employed as well geographic location, processing conditions, and genetic differences.

It is well known that catechins mainly confer an astringent taste to tea, while CAF offers a bitter taste. Catechins have been regarded as the major phenols in almost all kinds of teas, and in Azorean tea samples were they were eluted in the order of EGC, C, EC, EGCG, and ECG. Furthermore, some authors have reported that the levels of total and individual catechins show high seasonal fluctuations in reaction to temperature, irradiance, UV doses, and day length [25,26]. According to Tounekti et al. [27], tea is to be grown in temperate climates, and this influence should be taken into account in the future when seeking to optimize and/or maximize their catechin content. The level of EGC in spring zone 1 (8.14 mg/g DW) was higher than in summer (traces), and the same pattern was also observed for zone 2 (10.25 and 6.25 mg/g DW) for spring and summer, respectively. These fluctuations were also observed by Sharma et al. [26], who recorded higher EGC content, mostly during the growth flushes (June–September) when the hours with sunlight were longer, which increases the temperature and consequently leads to the biosynthesis of the catechins. These seasonal fluctuations are more critical in the Azores Islands, which are prone to large climate differences from one week to the next (it is normal to have four seasons in one day). It should also be pointed out that several studies that focused on the variation in tea catechins among different cultivars reported that geographic location, genetic variation, and agricultural practices have a significant effect on catechin levels [28]. Additionally, the impact of the growing environments (including soil types, soil fertility, temperatures, sunlight intensity, water stress, rainfall distribution, and growth altitude) have an effect on a tea’s composition, and consequently on its quality [27,29], and thus offers a possible explanation for the higher values of ECDs observed in green tea from Azorean samples. Furthermore, it should also be highlighted that comparing data from several studies is quite difficult given that different raw materials, extraction protocols, analytical methods, and units of measurement are used [3].

**Figure 3:**
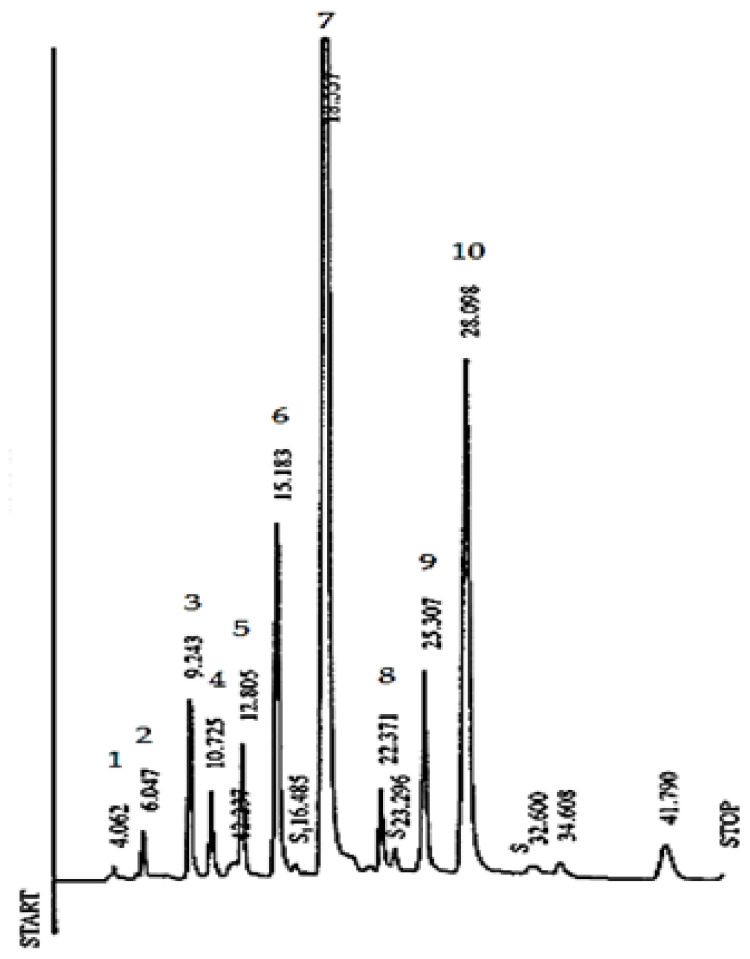
Reverse-phase HPLC of Azorean (Gorreana) tea catechins on a 5 µm Spherisorb ODS2 column (100 × 4.6 mm i.d.). Inj. vol. 10 µL. Mobile phase: acetonitrile:ethyl acetate:0.1% phosphoric acid:water (4.25:1:44.75:50, *v*/*v*/*v*/*v*)—phase (A), during 10 min, followed by a linear gradient between (A) and acetonitrile:water (1: 1, *v*/*v*)—phase (B), reaching 20% (B) in 10 min. Detection by UV (280 nm). Peaks: 1—GA, gallic acid; 2—GC, gallocatechin; 3—EGC, epigallocatechin; 4—C, catechin; 5—CAF, caffeine; 6—EC, epicatechin; 7—EGCG, epigallocatechin-3-*O*-gallate; 8—CA, p-coumaroylquinic acid; 9—GCG, gallocatechin-3-*O*-gallate; 10—ECG, epicatechin-3-*O*-gallate.

In the present study, and using the chromatographic conditions described in the methods section, the individual catechins and CAF were separated in a total run time of approximately 34 min, with a good separation of EC and EGCG, as illustrated in the chromatogram of the black tea sample (Figure 3).

### 3.2. Determination of Theaflavin Content Profiles and their Variability in Different Zones of Tea Plantation and in Different Seasons

**Figure 4:**
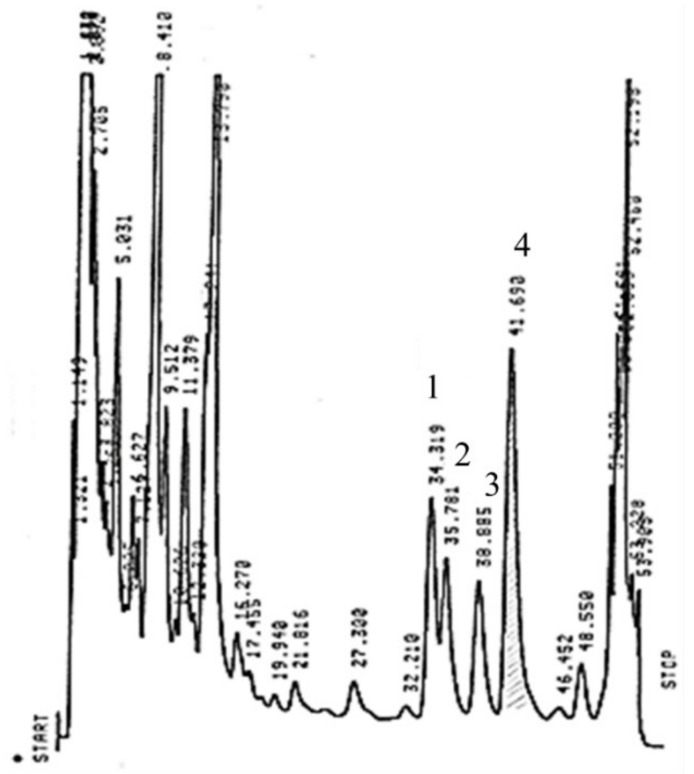
Reverse-phase HPLC of Azorean (Gorreana) tea on a 5 µm Hypersil ODS (100 × 4.6 mm i.d.). A 12.5 µL sample of the extract was subjected to HPLC analysis. Mobile phase: H_2_O:formic acid (99.7:0.3, *v*/*v*)—phase (A), and methanol:formic acid (99.7:0.3, *v*/*v*—phase (B). The separation was achieved by a linear gradient in the following conditions: t_0_ = 30% (B), t_6_ = 30% (B), t_40_ = 39% (B), and t_50_ = 60% (B) at a flow rate of 1 mL/min. Detection by UV (365 nm). Peaks: 1—TF, theaflavin; 2—TF-3-G, theaflavin-3-*O*-gallate; 3—TF-3′-G, theaflavin-3′-*O*-gallate; 4—TF-3,3′-DG, theaflavin-3,3′-di-*O*-gallate.

During the oxidation/fermentation process, due to the enzymatic action of PPO or POD, catechins are degraded into the B ring fission and polymerize into theaflavin and thearubigin derivatives [11]. It is well known that theaflavin levels are known to be related to the quality and taste of the black tea [7]. Using the chromatographic conditions described in the methods section, all theaflavin compounds were separated in a total run time of 50 min, with a good separation of TF-3′-G and TF-3,3′-DG. A standard mixture of four theaflavins were eluted in the following order: TF, TF-3-G, TF-3′-G, and TF-3,3′-DG. The higher concentrations of theaflavin components in black tea leaves are most likely a result of the complete fermentation process, where most catechins are converted into theaflavins [17]. Figure 4 presents a chromatogram obtained from a summer black tea extract. The peaks of the theaflavins presented longer RT with respect to those of the catechins, suggesting that shortening elution gradients could be utilized if only theaflavins are identified. Some authors use a simultaneous separation of catechins plus theaflavins in a long-run separation, but in this study, the authors used different chromatographic conditions for the separation of catechins and theaflavins, respectively, obtaining an improved resolution of the tea components.

The analytical parameters of the developed HPLC method for the TF-3,3′-DG determination are shown in Table 2 and were evaluated in terms of linearity, square of regression coefficient, LOD, LOQ, recovery, and reproducibility. The TF-3,3′-DG standard was spiked into the blank sample (2 mg/L in water) for the measurement of LOD and LOQ, which were similar to those reported by Matsubara and Rodriguez-Amaya [22]. The results showed that the relative standard deviation (RSD) for TF-3,3′-DG ranged between 0.06 and 0.57%, the reproducibility (inter-day and intra-day) RSD was < 0.6%, and a good level of recovery (95.8–104.8 %) was achieved.

Table 1 also shows the black tea samples for theaflavin contents in different seasons and different zones of the Gorreana Tea Plantation. TF-3,3′-DG is the galloylated theaflavin that has been reported to have a higher inhibition activity against SARS-CoV-2 3CL^pro^ [5].

The results showed that theaflavins contents in different tea samples changed according to the different zones and different seasons, similarly to the results reported by Jiang et al. [29]. We found that total theaflavins were higher in zone 3, followed by zone 1 and zone 2. According to the seasons, higher values were found in the summer season than in spring, with values ranging from 8.05 to 27.98 mg/g of DW in summer and 6.54 to 15.70 mg/g of DW in spring. The same pattern was also observed for all the theaflavins studied, with zone 3 showing higher values in the summer season. The highest value of TF was observed in summer, in zone 3 (6.43 mg/g of DW), and the lowest value in spring, in zone 2 (1.75 mg/g of DW). For TF-3-G, the values ranged from 1.99 to 5.39 mg/g of DW for summer in zones 2 and 3, respectively. Concerning TF-3′-G, the values ranged from 1.56 to 7.25 mg/g of DW for spring in zone 2 and summer in zone 3. In relation to TF-3,3′-DG, the highest values were observed in zone 3, with values of 3.72 and 8.91 mg/g of DW, followed by zone 1, with values of 2.68 and 3.78 mg/g of DW, and zone 2, with values of 1.13 and 2.06 mg/g of DW, for spring and summer, respectively. The highest values observed in zone 3, as already discussed for the catechins, can be explained by the higher altitude (360 m from the sea level) of the tea plantation and consequently by the different climatic conditions. Takemoto and Takemoto [30] revealed that several studies focusing on tea catechin and theaflavin variations among different cultivars reported that geographic location, genetic variation, and agricultural practices have a significant effect on catechin and theaflavins levels. According to German et al. [31], the concentrations of carbon and nitrogen in the soil, as well as soil pH, were most related to the hydrolytic extracellular enzyme activities that are negatively correlated with soil pH. Most hydrolytic active sites are optimal around pH 5, which can be correlated with the altitude of some tea plantation zones, influencing the PPO activity and consequently the formation of theaflavins.

According to Storozhuk [32], *C. sinensis* tea constituents could reduce overall risks related to COVID-19, and the results (see Table 3) clearly revealed a low percentage of infections by SARS-CoV-2 in countries with a higher consumption of *C. sinensis* tea. This implies that tea catechins and theaflavins may be effective in the prevention/treatment of COVID-19 or the amelioration of its severity [5,6,33,34,35]. These results may explain the reason for a lower number of deaths per million of population in countries with a high tea consumption as compared with countries with a lower tea consumption.

### 3.3. Variability of TF-3,3′-DG in Different Seasons, in Different Parts of the Tea Plant, and in Different Fermentation Times and the Effect of Fermentation Temperature on TF-3,3′-DG Content from Different Zones of Tea Plantation

**Figure 5:**
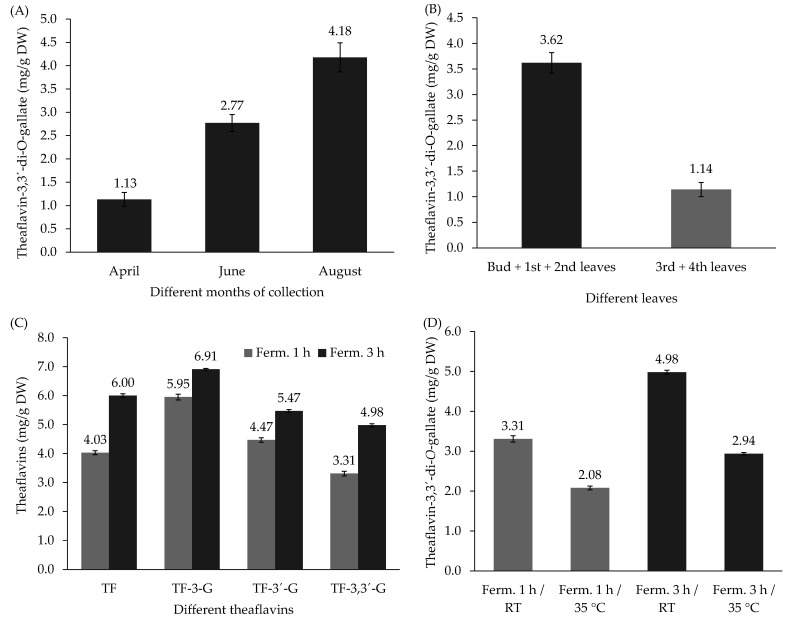
(**A**) Different theaflavin-3,3′-di-*O*-gallate content in the same zone but from different months; (**B**) different theaflavin-3,3′-di-*O*-gallate content in the same zone but from different parts of the tea plant; (**C**) variability of different theaflavins contents according to different fermentation times (1 h vs. 3 h); (**D**) the effect of room temperature (RT) vs. 35 °C on theaflavin-3,3′-di-*O*-gallate content from the same tea plantation zone and at different fermentation times (1 h vs. 3 h).

Figure 5A shows the different TF-3,3′-DG content in the same tea plantation zone but from different plucking months, April, July, and August, which presented the values of 1.13, 2.77, and 4.18 mg/g DW, respectively, showing higher values in summer with respect to spring. These results can be explained by the higher rate of tea plant metabolism during the warmer months. Zhao et al. [12] reported significantly lower values for theaflavins with respect to our results, which may be related to differences in terms of the extraction/analysis methodologies employed as well as genetic differences, geographic location, processing, and storage conditions.

Figure 5B shows different TF-3,3′-DG content in the same tea plantation zone, but from different parts of the tea plant, revealing higher values in the bud and first and second leaves (3.62 mg/g DW) with respect to the content in the third and fourth leaves (1.14 mg/g DW). It is well known that the catechin content, and consequently the theaflavin contents, decreases with the leaf’s age, showing the highest content in the bud and first leaf.

Figure 5C illustrates the variability of different theaflavin contents according to the fermentation time (1 h vs. 3 h) at the same room temperature (20–22 °C). The results highlighted different contents due to the different fermentation times. When increasing the fermentation time from 1 to 3 h, all theaflavin contents increased (TF increased from 4.03 to 6.00, TF-3-G increased from 5.95 to 6.91, TF-3′-G increased from 4.47 to 5.47, and TF-3,3′-DG increased from 3.31 to 4.98 mg/g DW).

The impact of fermentation temperature (room temperature vs. 35 °C) on TF-3,3′-DG content in the same tea plantation zone at different fermentation times (1 vs. 3 h) is presented in Figure 5D. The results revealed that the fermentation of *C. sinensis* leaves at room temperature presented higher values of TF-3,3′-DG with respect to 35 °C at the same fermentation time and at 3 h of fermentation presented the highest values. These results can be explained by the instability of theaflavins at higher temperatures [30] and the effect of PPO and POD on TF-3,3′-DG content that increased with fermentation time [31]. According to Ahmed et al. [28], environmental factors including temperature, sunlight intensity, water stress, rainfall distribution, and growth altitude variably impact the secondary metabolites produced in tea and have an effect on the tea’s uniqueness [7]. These facts offer a possible explanation for the higher values of theaflavins in black tea from different Azorean tea plantation zones.

## 4. Conclusions

The theaflavins from Azorean black tea were well separated by liquid chromatography (HPLC) in a reverse-phase C18 column. The limits of detection and quantification were in the range of 0.16–0.20 and 0.40–1.08 mg/L, respectively, and the recovery was in the range of 95.8–104.8%.

The EGCG content in Azorean black tea was higher in spring than in summer samples for all zones, and the contents of total catechins showed that spring samples from zone 2 presented higher values than in summer samples, and conversely, zones 1 and 3 presented higher values in summer.

The TF-3,3′-DG content in Azorean black tea was also affected by the different processing/climatic conditions, as summarized below.

The TF-3,3′-DG from the same season, but from different tea plantation zones, presented different contents, with the highest values observed in the higher altitude zone 3 (360 m from the sea level).

The TF-3,3′-DG from the different plucking months showed higher contents in summer months.

Higher contents of TF-3,3′-DG were observed in the bud and the first and second leaves as compared with contents in third and fourth leaves.

The TF-3,3′-DG content slightly increased with increasing fermentation time from 1 to 3 h and presented higher values at room temperature fermentation as compared with 35 °C.

This study revealed the possibility of creating a novel Azorean antiviral tea, investigating the black tea’s processing conditions to maximize the TF-3,3′-DG content as an inhibitor of 3CL^pro^ enzyme and, consequently, reducing the SARS-CoV-2 infectivity as already reported by several research teams.

## Figures and Tables

**Table 1:** Catechins, caffeine, and theaflavin contents in different tea plantation zones (mg/g dry weight) ^1^.

Parameter	Compound	Zone 1		Zone 2		Zone 3	
		Spring	Summer	Spring	Summer	Spring	Summer
Catechins	EGC	8.14 ± 0.41 ^b^	tr ^b^	10.25 ± 0.51 ^a^	6.25 ± 0.31 ^a^	tr ^c^	tr ^b^
	C	5.76 ± 0.10 ^a^	6.07 ± 0.09 ^a^	3.94 ± 0.06 ^c^	6.69 ± 0.09 ^a^	4.38 ± 0.08 ^b^	4.77 ± 0.11 ^b^
	EC	20.36 ± 1.12 ^a^	17.80 ± 0.95 ^c^	18.37 ± 1.06 ^b^	26.55 ± 1.42 ^a^	14.62 ± 0.66 ^c^	24.07 ± 1.01 ^b^
	EGCG	67.01 ± 1.17 ^c^	54.03 ± 1.12 ^b^	94.71 ± 1.01 ^b^	54.23 ± 1.39 ^b^	133.64 ± 1.80 ^a^	121.30 ± 3.44 ^a^
	ECG	73.47 ± 1.15 ^a^	105.69 ± 2.28 ^a^	73.63 ± 1.27 ^a^	97.64 ± 1.52 ^ab^	59.11 ± 0.73 ^b^	64.95 ± 1.83 ^c^
	TOTAL	174.72 ^c^	183.59 ^c^	200.90 ^ab^	191.36 ^b^	211.75 ^a^	215.09 ^a^
Caffeine		16.12 ± 0.54 ^a^	16.18 ± 1.03 ^a^	13.67 ± 0.48 ^b^	12.27 ± 0.29 ^c^	6.40 ± 0.07 ^c^	14.15 ± 0.38 ^b^
Theaflavins	TF	3.29 ± 0.12 ^b^	3.41 ± 0.21 ^b^	1.75 ± 0.06 ^c^	1.90 ± 0.12 ^c^	4.04 ± 0.13 ^a^	6.43 ± 0.40 ^a^
	TF-3-G	3.99 ± 0.18 ^a^	4.22 ± 0.19 ^b^	2.10 ± 0.06 ^b^	1.99 ± 0.08 ^c^	4.16 ± 0.18 ^a^	5.39 ± 0.16 ^a^
	TF-3′-G	2.45 ± 0.09 ^b^	3.45 ± 0.15 ^b^	1.56 ± 0.11 ^c^	2.10 ± 0.16 ^c^	3.78 ± 0.11 ^a^	7.25 ± 0.25 ^a^
	TF-3,3′-DG	2.68 ± 0.11 ^b^	3.78 ± 0.07 ^b^	1.13 ± 0.15 ^c^	2.06 ± 0.25 ^c^	3.72 ± 0.09 ^a^	8.91 ± 0.40 ^a^
	TOTAL	12.41 ^b^	14.86 ^B^	6.54 ^c^	8.05 ^C^	15.7 ^a^	27.98 ^A^

^1^ Values are mean ± SD (*n* = 3). Different superscript letters in the same seasons and in the same row are significantly different (*p* < 0.05). tr—traces; C—catechin; EC—epicatechin; ECG—epicatechin-3-*O*-gallate; EGC—epigallocatechin; EGCG—epigallocatechin-3-*O*-gallate; TF—theaflavin; TF-3-G—theaflavin-3-*O*-gallate; TF-3′-G—theaflavin-3′-*O*-gallate; TF-3,3′-DG—theaflavin-3,3′-di-*O*-gallate.

**Table 2:** Analytical parameters of the developed HPLC method.

Analyte	Linear Range (mg/L)	R^2^	LOD (mg/L)	LOQ (mg/L)	Recovery (%)	Inter-Day/Intra-Day (% RSD)
TF-3,3′-DG	0.18–94.0	0.9998	0.16–0.20	0.40–1.08	95.8–104.8	0.57/0.06

R^2^–Square of regression coefficient; RSD—Relative standard deviation (*n* = 5); LOD—limit of detection (inj. vol. = 12.5 µL); LOQ—limit of quantification (inj. vol. = 12.5 µL).

**Table 3:** Countries with “low” and “high” (above 150 g per/capita) tea consumption relative to the total COVID-19 mortality per millions of people and percentage of COVID-19 mortality per total infections in different countries.

Country	Population in Millions	Total COVID-19 Cases ^1^	Total COVID-19 Mortality ^1^	COVID-19 Mortality (per Million Population) ^1^	% COVID-19 Mortality per Total Cases ^2^	% COVID-19 Cases per Million Population ^1^
Low Tea Consumption Countries						
Germany	83.13	7,279,037	112,707	1356	1.55	0.87
France	67.51	10,694,804	125,551	1860	1.17	15.84
Italy	59.09	6,566,947	138,045	2336	2.10	11.11
Spain	47.39	6,785,286	89,689	1893	1.32	14.32
Portugal	10.51	1,460,406	19,015	1809	1.30	13.89
High Tea Consumption Countries						
China	1439.32	115,611	4849	3.37	4.19	0.08
Japan	126.93	1,735,050	18,393	144.91	1.06	1.37
Senegal	16.74	77,935	1895	113.20	2.43	0.47
Korea	51.67	645,226	5781	111.88	0.89	1.25
Kenya	47.56	302,134	5401	113.56	1.79	0.64
Mozambique	31.26	196,346	2042	65.32	1.04	0.63
Taiwan	23.83	17,129	849	35.63	4.96	0.07
Uzbekistan	33.47	199,758	1488	44.46	0.74	0.60
Mali	20.25	23,600	670	33.09	2.84	0.12
Other Tea Consumption Countries						
Canada	38.51	2,323,772	30,475	791.14	1.31	6.03
USA	332.90	56,803,703	829,351	2491	1.46	1.71
Brazil	212.56	22,309,081	619,473	2914	2.78	10.50

^1^ Data from Johns Hopkins Coronavirus Resource Center [36]. ^2^ Higher ratio means low support by Health Care Institutions for COVID-19 patients.

## Data Availability

All data are contained within the article.
